# Investigation of the Phosphorus Effect on Solidification Cracking in Cu–Steel Single-Mode Fiber-Laser Welds for Reliable Li-Ion Battery Busbar Assembly

**DOI:** 10.3390/ma18245585

**Published:** 2025-12-12

**Authors:** Ye-Ji Yoo, Jeong-Hoi Koo, Eun-Joon Chun

**Affiliations:** 1 Department of Materials System Engineering, Pukyong National University, Busan 48513, Republic of Korea; 2 Department of Mechanical and Manufacturing Engineering, Miami University, Oxford, OH 45056, USA

**Keywords:** single mode fiber laser, Cu–steel dissimilar welding, solidification cracking, phosphorus, mushy zone range

## Abstract

Solidification cracking is a critical defect in Cu–steel dissimilar laser welding for cylindrical lithium-ion battery busbar assembly, yet the metallurgical role of phosphorus (P) in crack formation has not been quantitatively established. In this study, the influence of phosphorus in the coating layer on weld solidification behavior was clarified by preparing Cu substrates with four different coating conditions—Ni–P-coated Cu (10 and 50 μm) and pure Ni-coated Cu (10 and 50 μm)—and performing high-speed single-mode fiber-laser welding under identical heat-input conditions. Shear-tensile testing, EPMA-based microstructural analysis, and Thermo-Calc solidification calculations were combined to correlate P segregation with solidification cracking susceptibility. The Ni–P 10 μm coating generated severe solidification cracking compared with the pure Ni 50 μm coating, which was attributed to excessive P enrichment in the terminal liquid phase (up to 8.8 mass%). This enrichment significantly expanded the mushy-zone width to approximately 869 K, yielding a highly solidification crack-susceptible fusion zone. In contrast, 50 μm pure Ni coatings produced narrow mushy-zone widths (200–400 K) and extremely low residual P levels (~0.1 mass%), resulting in fully crack-free microstructures. The 50 μm Ni coating exhibited the highest shear-tensile strength and largest rupture displacement among all conditions, confirming that suppression of P segregation directly improves both structural integrity and mechanical performance. Overall, this study demonstrates that phosphorus enrichment critically governs the solidification-cracking susceptibility of Cu–steel dissimilar welds by widening the solidification temperature range. Eliminating P from the coating layer and applying an adequately thick pure Ni coating constitute highly effective strategies for achieving crack-free, mechanically robust welds in lithium-ion battery busbar manufacturing.

## 1. Introduction

The accelerating global transition toward electric vehicles (EVs), driven by stringent environmental regulations, has created a growing demand for high-performance and durable lithium-ion battery systems [1,2,3]. Each battery pack consists of thousands of interconnected cells, and the quality of the welded joints—particularly in Cu–steel dissimilar connections—directly determines the electrical reliability and mechanical robustness of the entire module [4,5,6]. In cylindrical battery cells, steel is commonly used for electrode terminals, while copper (Cu) serves as the busbar material owing to its superior electrical conductivity [7,8,9]. Consequently, Cu–steel dissimilar welding has become a key joining technology in EV battery manufacturing. Despite remarkable advances in laser welding technologies, solidification cracking remains one of the most critical issues in Cu–Fe dissimilar welds [10,11,12]. This difficulty arises from the large mismatch in thermal conductivity, melting temperature, and solidification behavior between Cu and Fe, which induces severe segregation and thermal stress during rapid solidification [13,14,15,16,17]. Recent studies employing blue and single-mode fiber lasers have consistently reported the formation of hot cracks in Cu–Fe welds, emphasizing the need for a more detailed understanding of the underlying metallurgical mechanisms [18,19,20,21,22]. Recent advances in welding research have increasingly incorporated numerical simulations, machine-learning-assisted process optimization, and real-time acoustic emission monitoring to enhance laser and hybrid weld quality [23,24,25]. These trends collectively reinforce the importance of analyzing defect-sensitive solidification behavior under high-speed processing conditions, which remains a key metallurgical challenge in Cu–steel dissimilar busbar welding.

In our earlier study [26], high-speed single-mode fiber laser welding (>1000 mm/s) was applied to Cu–SS275 dissimilar joints to systematically examine their solidification behavior. The results revealed that cracking occurred mainly in localized regions exhibiting a wide solidification temperature range (mushy zone) of approximately 450 K, accompanied by pronounced Cu segregation. Furthermore, introducing an electroless Ni–P coating of about 10 μm on the Cu substrate, as compared with the bare Cu (uncoated) condition, resulted in a relative reduction in solidification cracking. This improvement was attributed to the presence of Ni, which reduced solute segregation and narrowed the solidification temperature range. However, when the coating thickness exceeded this range, cracking reappeared—presumably due to phosphorus enrichment at the fusion boundary, which broadened the mushy zone and locally weakened the microstructure. These observations suggest that Ni and P exert contrasting metallurgical influences during weld solidification. While Ni tends to promote compositional uniformity and stabilize the solid–liquid interface, phosphorus may broaden the solidification interval and promote crack formation by altering phase equilibria [27,28,29,30,31,32,33,34]. Representatively, phosphorus (P) enrichment is widely recognized as a primary contributor to solidification-crack susceptibility in steel welds, often in conjunction with sulfur (S). In stainless steels, under high-speed or rapid solidification conditions, even modest increases in P + S significantly widen the solidification brittle temperature range by promoting segregation into interdendritic terminal liquid, where low-melting films can persist and delay final solidification, thereby expanding the mushy zone [35]. TRIP steel weld studies have directly confirmed intense P partitioning into crack-adjacent interdendritic liquid, stabilizing transient liquid films that coincide with crack initiation sites [36]. Similar mechanisms involving low-melting boundary-liquid stabilization and consequent mushy-zone broadening have been reported in Ni–Cr–Fe-based alloy weld metals [37]. In this regard, the influence of phosphorus in the coating layer on solidification cracking during Cu–steel busbar welding remains unclear, and previous studies have not sufficiently provided a clear comparison of cracking behavior based on the presence or absence of phosphorus.

Therefore, the present study was designed as a continuation of our previous work, focusing on a comparative analysis of solidification-cracking behavior depending on the presence or absence of phosphorus within the coating layer during Cu–steel dissimilar welding. To achieve this, the same electroless Ni–P coating condition used in the previous investigation was employed, together with a pure Ni coating as a comparative reference, under identical single-mode fiber laser parameters. In addition, the experimental findings are further supported and theoretically examined through thermodynamic calculations, allowing a more comprehensive understanding of how phosphorus influences the solidification temperature range, microstructural evolution, and crack susceptibility in Cu–steel dissimilar welds.

## 2. Materials and Methods

### 2.1. Materials

High-purity copper (C1100P, 100 × 30 × 0.5 mm) and mild steel (SS275, 100 × 30 × 0.5 mm) were used as base materials, identical to those employed in the previous study. The corresponding chemical compositions are provided in Table 1. To examine the solidification cracking behavior depending on the presence or absence of phosphorus, two types of coatings were prepared on the Cu substrate: electroless Ni–P coatings and electroplated pure Ni coatings. Each coating was applied symmetrically on both upper and lower surfaces of the Cu sheet. Three coating-thickness conditions were examined: uncoated (0 μm), 10 μm, and 50 μm. The electroless Ni–P layer was deposited from an acidic NiSO_4_ bath at 85–95 °C without applied current, while the pure Ni coating was electrodeposited using a nickel sulfamate and boric acid bath at 3–8 A/dm^2^. These coating method conditions are summarized in Table 2.

### 2.2. Laser Welding Procedure

**Figure 1:**
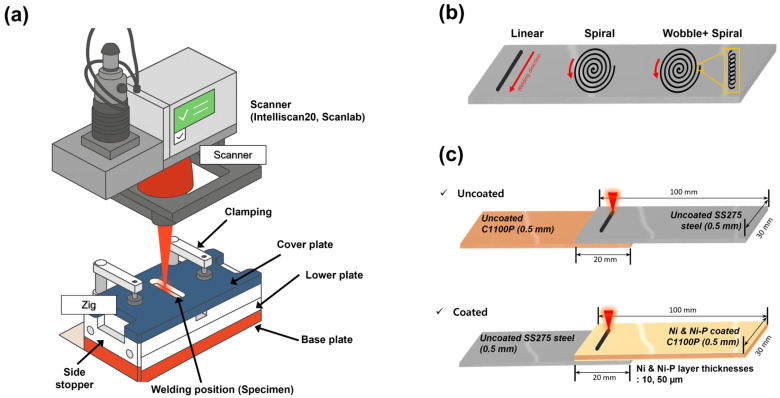
Visual and schematic descriptions of (**a**) the laser-welding experiment and (**b**) beam-scanning patterns, and (**c**) the shear–tensile test specimen configuration.

Figure 1 schematically illustrates the experimental setup, beam-scanning patterns, and the geometry of the shear-tensile-test specimens. Laser welding was performed using a single-mode fiber laser (YLS-2000-SM, IPG Photonics, Oxford, MA, USA) equipped with a high-speed scanning head (IntelliScan 20, Scanlab, Puchheim, Germany). The laser operated at a wavelength of 1068 nm with a maximum power of 2 kW and a focal-spot diameter of 38 μm. The beam was focused on the specimen surface, and the scan speed was maintained at 1100 mm s^−1^, corresponding to a heat input of approximately 1.82 J mm^−1^. Three beam-scanning patterns—Linear, Spiral, and Wobble + Spiral (4000 Hz Wobble frequency, 0.14 mm Wobble amplitude, and 0.28 mm pitch)—were employed (Table 3). All laser-welding parameters were intentionally kept identical to those used in our previous study to ensure direct comparability of the results and to isolate the metallurgical influence of the coating composition.

### 2.3. Mechanical and Microstructural Characterization

The mechanical performance of the welds was evaluated using a shear-tensile test conducted at a crosshead speed of 3 mm min^−1^. For each coating condition of Ni and Ni–P (0, 10, and 50 μm), seven repeated tests were performed, and the average maximum load and displacement were calculated after excluding the highest and lowest values for each coating thickness and beam-scanning pattern. This exclusion was applied to eliminate potential outliers and improve the robustness of the averaged results. Standard deviations and 95% confidence intervals were calculated for all coating and beam-pattern conditions to ensure the statistical reliability of the measured mechanical properties.

Microstructural analysis was performed using an optical microscope (OM, Leica DR IRM, Wetzlar, Germany) and electron probe microanalysis (EPMA, JEOL JXA-8530F, Tokyo, Japan) to examine elemental distributions and segregation behavior near the fusion boundary. Particular attention was paid to detecting localized P-enriched zones, as these regions were previously inferred to correlate with solidification crack initiation.

## 3. Results and Discussion

### 3.1. Comparison of Solidification Cracking Behavior in Ni–P and Ni-Coated Cu–Steel Welds

**Figure 2:**
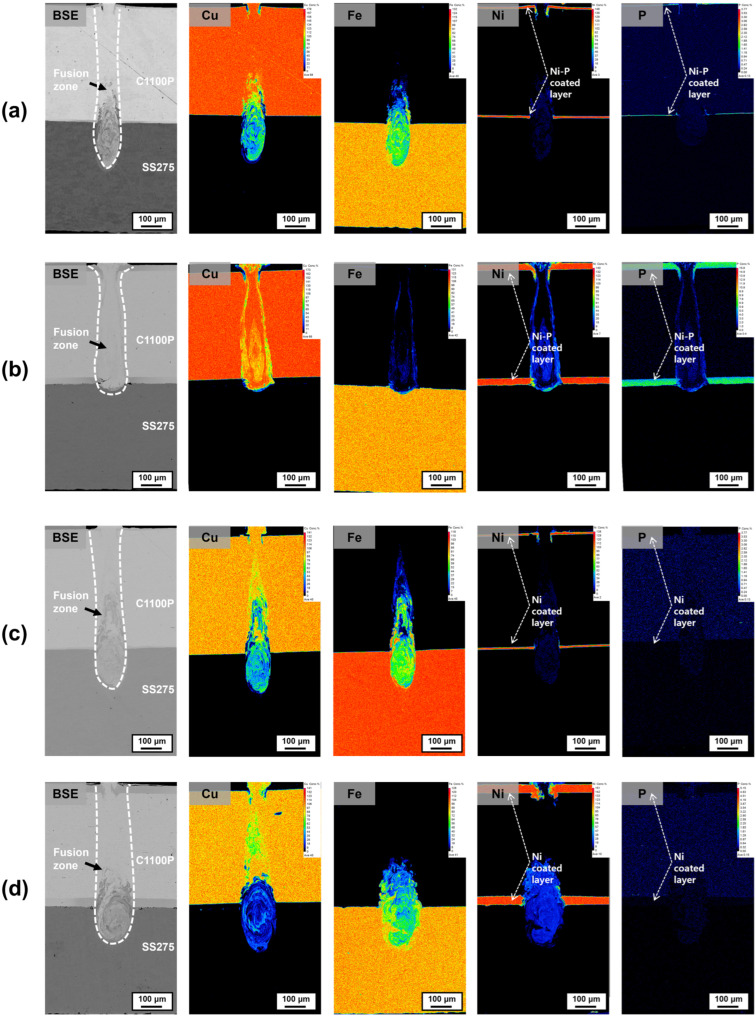
Elemental distribution maps of Cu–steel Linear welds produced using (**a**) 10 μm and (**b**) 50 μm Ni–P coatings, and (**c**) 10 μm and (**d**) 50 μm pure Ni-coatings.

Figure 2 shows the backscattered-electron (BSE) images and EPMA elemental distribution maps of Cu–steel (SS275) dissimilar welds fabricated using a single-mode fiber laser under the Linear beam-scanning pattern. The analyzed specimens include (a) Ni–P 10 μm coated, (b) Ni–P 50 μm coated, (c) pure Ni 10 μm coated, and (d) pure Ni 50 μm coated Cu–steel welds, respectively. In all cases, distinct coating layers were observed on both upper and lower surfaces of the Cu substrate. Phosphorus peaks were clearly detected in the Ni–P-coated specimens (Figure 2a,b), whereas only Ni signals appeared in the pure Ni-coated specimens (Figure 2c,d), confirming that high-purity Ni layers were successfully deposited on Cu. Due to the characteristics of the single-mode fiber laser, all conditions produced narrow and deep keyhole-shaped weld beads. Under the Linear pattern, the average penetration depth for the Ni–P-coated Cu–steel welds was 0.65 ± 0.032 mm (with approximately 30% dilution of the lower steel), and the bead width was 0.14 ± 0.012 mm. Similarly, for the Ni-coated welds, the average penetration depth and bead width were 0.64 ± 0.041 mm and 0.15 ± 0.018 mm, respectively, confirming that both coating types produced nearly identical weld geometries.

**Figure 3:**
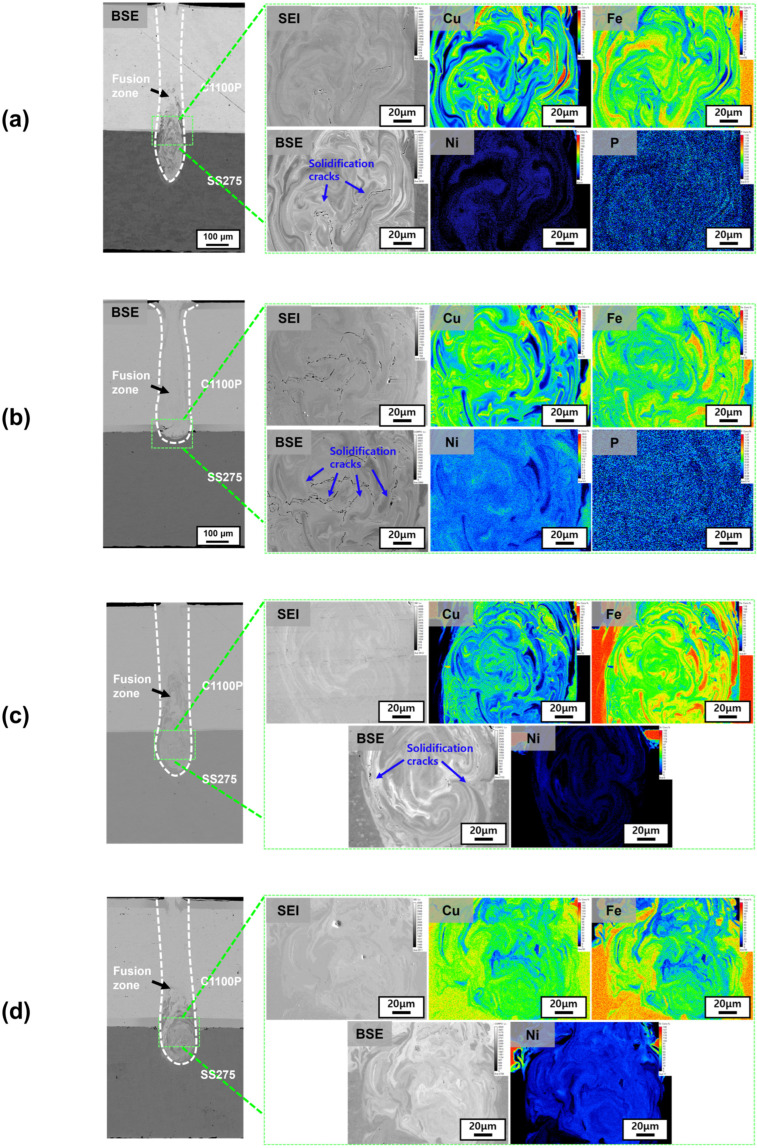
Element distribution and enlarged view for solidification cracking site for Cu–steel Linear welds; using (**a**) 10 μm and (**b**) 50 μm Ni–P coated, (**c**) 10 and (**d**) 50 μm of pure Ni-coated Cu.

Figure 3 presents high-magnification EPMA analysis results for the Ni- and Ni–P-coated Cu–steel (SS275) welds. Compared with the lower-magnification BSE images in Figure 2, micro-scale solidification cracks were newly identified that were not visible under the low magnifications. This finding indicates that in high-speed single-mode fiber-laser welding of Cu–steel (SS275) dissimilar materials, micro-cracks can form at the micrometer scale even when the weld surface appears macroscopically sound. Such micro-level cracking, though difficult to detect at low magnification, must be strictly controlled in precision manufacturing fields requiring high-integrity micro-welds, such as the Li-ion battery busbar welding process. Consistent with the previous study [26], the Ni–P-coated specimens exhibited slightly suppressed cracking behavior at a 10 μm coating thickness (Figure 3a) but showed pronounced solidification cracking at 50 μm (Figure 3b). In contrast, the pure Ni-coated specimens displayed crack-free microstructures, especially at both 50 μm (Figure 3d) coating thicknesses, indicating that solidification cracking was effectively suppressed regardless of layer thickness. These observations demonstrate that the solidification-cracking behavior of Cu–steel dissimilar welds is strongly dependent on both the coating composition and its thickness. Specifically, phosphorus addition in the Ni–P coatings increases the susceptibility to micro-crack formation when the coating becomes excessively thick, whereas pure Ni coatings consistently yield a dense, crack-free fusion boundary. This microstructural trend clearly supports the conclusion that the presence and thickness of the coating layer critically control solidification crack formation in Cu–steel dissimilar laser welds.

**Figure 4:**
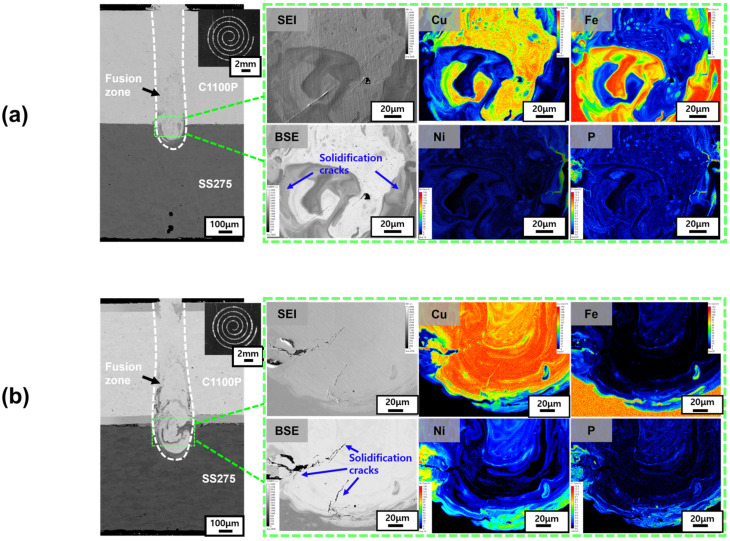
Elemental distribution maps of Ni–P-coated Cu–steel welds produced using the Spiral beam pattern: (**a**) 10 μm Ni–P coating; (**b**) 50 μm Ni–P coating.

**Figure 5:**
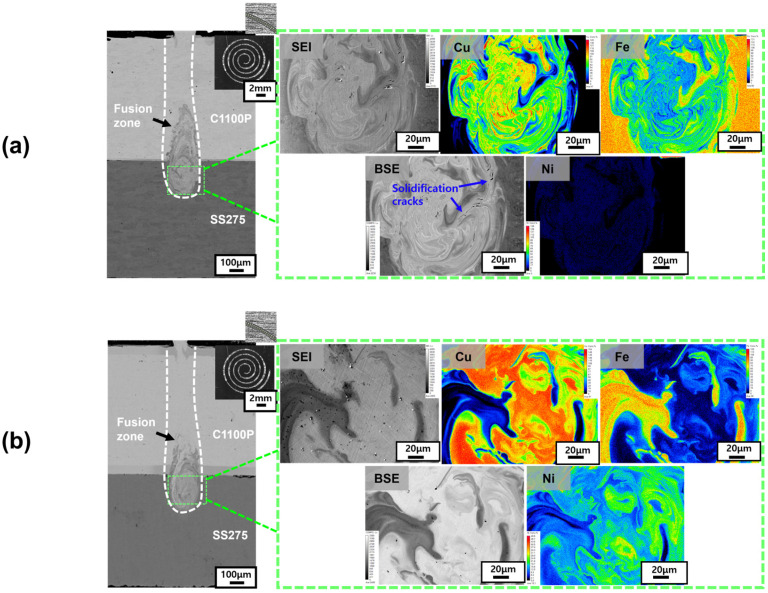
Elemental distribution maps of Ni–coated Cu–steel welds produced using the Wobble + Spiral beam pattern: (**a**) 10 μm Ni–P coating; (**b**) 50 μm Ni–P coating.

Figure 4 and Figure 5 show the EPMA analysis results of Spiral and Wobble + Spiral beam-scanning patterns for the Ni–P-coated (Figure 4a (10 μm), Figure 4b (50 μm)) and pure Ni-coated (Figure 5a (10 μm), Figure 5b (50 μm)) Cu–steel welds. The solidification-cracking tendencies observed under these scanning patterns were generally consistent with those obtained from the Linear pattern as described in Figure 3. For the Ni–P-coated specimens (Figure 4), a substantial increase in solidification cracking was observed as the coating thickness increased from 10 μm to 50 μm, whereas the pure Ni-coated specimens (Figure 5) exhibited minor cracking at 10 μm, but complete crack suppression was achieved at 50 μm of Ni coating thickness. This confirms that phosphorus enrichment within the coating layer significantly enhances crack susceptibility when the layer becomes excessively thick, while a thicker pure Ni coating stabilizes solidification and prevents crack initiation.

When comparing the results of the Linear (Figure 3), Spiral (Figure 4), and Wobble + Spiral (Figure 5) patterns, both Ni–P and Ni-coated Cu–steel welds exhibited solidification cracks mainly near the faying surface, where the coating elements were most heavily diluted into the fusion zone. In the Ni–P-coated welds, localized P enrichment corresponded precisely to regions where solidification cracks initiated, indicating that phosphorus segregation directly contributes to the formation of low-melting constituents and micro-scale cracking. In contrast, for pure Ni-coated welds, solidification cracking was consistently suppressed in all beam patterns, particularly at the 50 μm thickness condition.

Comprehensive analysis of all crack morphologies and EPMA maps indicates that, under identical beam conditions, the weld penetration depth, dilution ratio, and compositional homogeneity were nearly equivalent, and the dominant variable affecting crack formation was the type and thickness of the coating layer. Moreover, in these single-mode laser welds, no macroscopic defects such as pores or lack of fusion were observed in any of the examined weld sections for all laser beam patterns, confirming that the mechanical degradation was governed primarily by solidification cracking. Therefore, the mechanical behavior of the welds is expected to be strongly influenced by the degree of solidification cracking. Accordingly, Section 3.2 indirectly compares the solidification-crack susceptibility among coating conditions based on shear-tensile testing results.

### 3.2. Solidification-Cracking Susceptibility Indirectly Evaluated by Shear-Tensile Test

In continuation of the microstructural analysis, this section indirectly evaluates the solidification-cracking susceptibility of Cu–steel dissimilar welds through their shear-tensile performance. From the preceding observations, it was inferred that the shear-tensile strength of the welds is strongly dependent on the extent of solidification cracking. Accordingly, the tensile results were used as an indirect quantitative indicator of crack susceptibility. A total of 18 weld conditions were compared, combining three beam-scanning patterns (Linear, Spiral, and Wobble + Spiral) with three coating thicknesses (uncoated, 10 μm, and 50 μm) for both Ni–P and pure Ni coatings. In particular, the tensile properties for the uncoated and Ni–P-coated Cu specimens were adopted from our previous study [26], allowing direct comparison with the newly evaluated pure Ni-coated welds. Notably, irrespective of coating composition, thickness, or laser beam-scanning pattern, all weld specimens consistently fractured within the weld region. This consistency enables a direct comparison of mechanical performance within each beam pattern and allows the effects of coating conditions on solidification-cracking behavior to be indirectly assessed.

**Figure 6:**
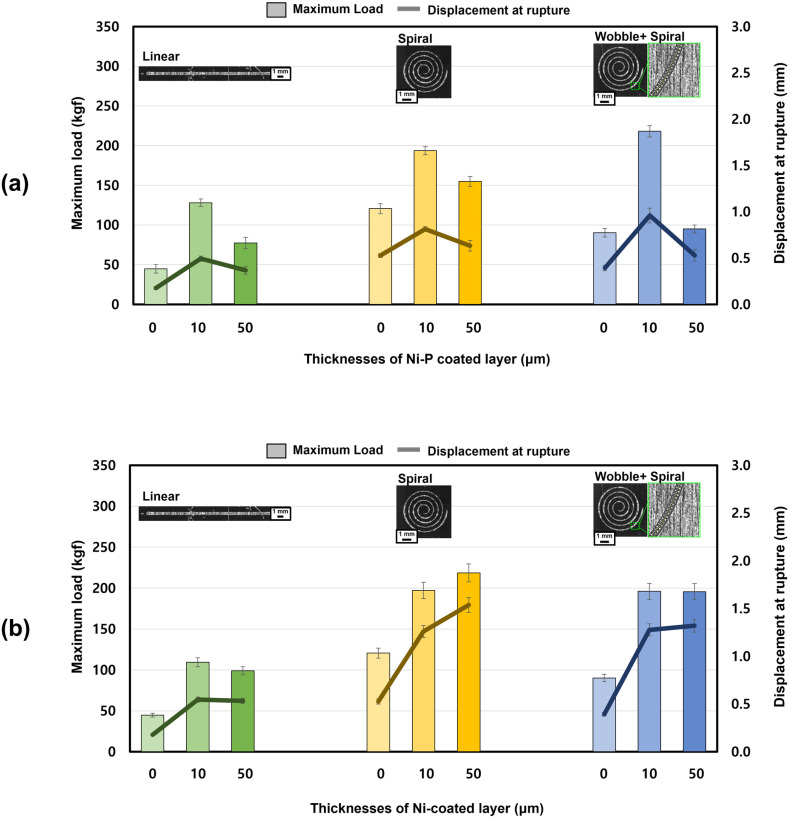
Effect of coating composition and thickness on the maximum load and displacement at rupture for (**a**) Ni–P-coated Cu–steel welds [26] and (**b**) pure Ni-coated Cu–steel welds.

Figure 6a shows that the maximum load and displacement at rupture of the Ni–P-coated Cu–steel welds exhibited an initial increase but then a sharp decrease as the coating thickness increased from 10 to 50 μm [26]. In contrast, Figure 6b reveals that the pure Ni-coated Cu–steel welds displayed a monotonic increase in both maximum load and displacement with increasing coating thickness for all laser beam-pattern conditions. Notably, the 50 μm Ni coating was the only condition among all coating types and thicknesses that produced a significant improvement in both load-bearing capacity and rupture displacement (Linear: 99 ± 5.93 kgf, 95% confidence intervals 93.5–104.5 kgf; Spiral: 219 ± 3.70 kgf, 95% confidence intervals 215.6–222.4 kgf; Wobble + Spiral: 190 ± 15.9 kgf, 95% confidence intervals 175.3–204.7 kgf). For other welding conditions, the standard deviation and 95% confidence intervals of shear-tensile strength were listed in Table 4. This behavior was not observed in any of the Ni–P-coated welds, indicating that the strengthening effect is unique to the phosphorus-free Ni coating, which is consistent with the complete suppression of solidification cracking in these welds.

### 3.3. Variation in Solidification Cracking Susceptibility via Non-Addition of Phosphorus Interpreted from Calculated Weld Mushy-Zone Range

**Figure 7:**
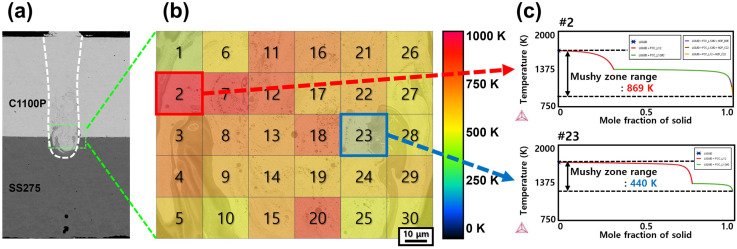
10 μm Ni–P-coated Cu–steel welds produced with the Linear beam pattern: (**a**) BSE image of the weld cross-section; (**b**) distribution map of the calculated mushy-zone range; and (**c**) representative solidification paths corresponding to the regions with the maximum and minimum mushy-zone range. # denotes the numbered locations, and the symbol represents the Thermo-Calc logo.

**Figure 8:**
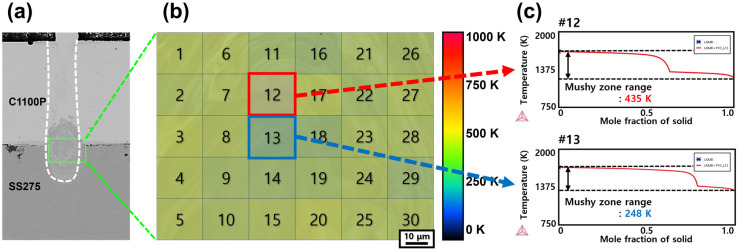
10 μm Ni-coated Cu–steel welds produced with the Spiral beam pattern: (**a**) BSE image of the weld cross-section; (**b**) distribution map of the calculated mushy-zone range; and (**c**) representative solidification paths corresponding to the regions with the maximum and minimum mushy-zone range. # denotes the numbered locations, and the symbol represents the Thermo-Calc logo.

**Figure 9:**
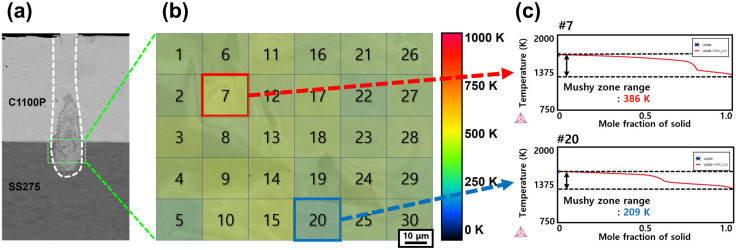
50 μm Ni-coated Cu–steel welds produced with the Spiral beam pattern: (**a**) BSE image of the weld cross-section; (**b**) distribution map of the calculated mushy-zone range; and (**c**) representative solidification paths corresponding to the widest and narrowest mushy-zone regions. # denotes the numbered locations, and the symbol represents the Thermo-Calc logo.

This section investigates the metallurgical factors governing the suppression and reduction in solidification cracking in Ni-coated Cu–steel welds compared with pure Ni–P-coated Cu–steel welds. Previous studies have reported that solidification cracking in Cu–steel dissimilar laser welds exhibits a strong correlation with the solidification temperature range of the fusion zone [26]. In the present study, this framework was extended to clarify the role of phosphorus in promoting solidification cracking by quantitatively examining how P influences the mushy-zone width of the weld metal. In earlier work, the solidification temperature range of the fusion zone was estimated under the assumption that the weld metal was uniformly diluted between the upper Cu and lower steel substrates, using the Rule of Mixtures to approximate the overall composition of the fusion zone, and the mushy-zone range was calculated based on these averaged compositions [26]. However, in the current study, to improve the accuracy of the discussion, the local chemical compositions were directly extracted from 30 representative positions within the fusion zone—specifically near the faying surface, where solidification cracking was most concentrated. These compositions were obtained through EPMA point analysis and subsequently used as input for calculating the local mushy-zone ranges using Thermo-Calc software (version 2025b). Table 5 summarizes the representative compositions used in these calculations. The mushy-zone calculations were performed for weld conditions exhibiting the highest and lowest solidification-cracking susceptibility, as determined from the mechanical test results in Figure 6. Accordingly, three representative cases were selected for detailed analysis:

(1) Ni–P 10 μm coated Cu–Steel welds with the Linear beam pattern (Figure 7),
(2) pure Ni 10 μm coated Cu–Steel welds with the Spiral beam pattern (Figure 8), and
(3) pure Ni 50 μm coated Cu–Steel welds with the Spiral beam pattern (Figure 9).

The calculation conditions used in Thermo-Calc are summarized in Table 6.

Figure 7 (Ni–P 10 μm coated Cu–steel welds with the Linear beam pattern), Figure 8 (pure Ni 10 μm coated Cu–steel welds with the Spiral beam pattern), and Figure 9 (pure Ni 50 μm coated Cu–steel welds with the Spiral beam pattern) present the calculated solidification temper-ature ranges obtained from a total of 30 measurement points within the rectangular region marked in Figure 7, Figure 8 and Figure 9a (area: 0.01 mm^2^) for each weld metal. From the previous analysis results (Figure 3), we could clearly confirm the faying surface, where cracks were concentrated. Since this area was specifically targeted for point analysis to calculate the weld mushy-zone range. The calculated mushy-zone range values were spatially mapped in Figure 7, Figure 8 and Figure 9b, where the mushy-zone ranges are visualized according to the following color scheme: 0–250 K (blue), 256–500 K (green), 501–725 K (yellow–orange), and 726–1000 K (red), representing progressively wider solidification temperature ranges. Figure 7, Figure 8 and Figure 9c show representative solidification paths corresponding to the measurement points that exhibited the maximum and minimum mushy-zone range within each mapping. It should be noted that for the Ni–P 50 μm coated Cu–steel welds, excessively high phosphorus concentrations in the cracking sites caused abnormal Thermo-Calc convergence behavior. Specifically, the convergence failure originated from an excessively high level of phosphorus enrichment (>0.43 wt%), locally concentrated within the 50 μm Ni–P weld metal. Under this condition, Thermo-Calc was unable to maintain a stable equilibrium pathway, and the solidification simulation diverged before reaching the terminal liquid–solid transition. As a result, the mushy-zone range for the Ni–P 50 μm coated Cu–steel weld could not be fully calculated, because the locally saturated P at the crack-susceptible region induced numerical divergence during equilibrium solidification computation.

Figure 7b shows that the Ni–P 10 μm coated Cu–steel welds exhibit a mushy-zone range with an average of approximately 700 K, predominantly represented by the red color scale. As shown in Figure 7c, the locally calculated mushy-zone range ranged from a maximum of 869 K to a minimum of 440 K, indicating a large variation of about 430 K within the analyzed solidification cracking site. In contrast, the pure Ni-coated welds—Ni 10 μm (Figure 8b) and Ni 50 μm (Figure 9b)—showed substantially narrower mushy-zone widths, with average values of approximately 300 K, representing a significant reduction compared with the Ni–P-coated welds. Specifically, the Ni 10 μm coated Cu–steel welds (Figure 8c) exhibited mushy-zone widths ranging from 435 K (maximum) to 248 K (minimum), while the Ni 50 μm coated Cu–steel welds (Figure 9c) showed an even narrower range, from 386 K (maximum) to 209 K (minimum). Both the absolute values and the degree of variation were markedly smaller than those of the Ni–P-coated condition. These differences in the mushy-zone width were found to originate from variations in phosphorus segregation at residual liquid at the terminal stage of weld solidification. In particular, for the Ni–P 10 μm coated Cu–steel welds (Linear beam pattern), the phosphorus concentration in the residual liquid phase at the solidification completion stage was calculated to reach approximately 8.8 mass%. In contrast, for the Ni 50 μm coated Cu–steel welds (Spiral beam pattern), the calculations indicated that only about 0.1 mass% of phosphorus remained segregated in the residual liquid phase. Therefore, to ensure the soundness of the coated Cu–steel dissimilar welds, the non-addition of phosphorus in the coating layer is strongly recommended. Furthermore, for both suppressing solidification cracking and enhancing the mechanical strength of battery busbar welds, the application of pure Ni coatings, along with an increase in coating thickness, is considered an effective metallurgical strategy.

## 4. Conclusions

In this study, a strategy for suppressing solidification cracking in Cu–steel laser welds was investigated in the context of cylindrical lithium-ion battery busbar fabrication using a high-speed single-mode IR fiber laser welding process. Particular emphasis was placed on comparing solidification-cracking behavior depending on the presence or absence of phosphorus (P) in the coating layer by evaluating Ni–P and pure Ni coatings under identical welding conditions, thereby extending the understanding reported in our previous work. The major findings of this study are summarized as follows:

Among the three beam-scanning patterns (Linear, Spiral, and Wobble + Spiral) and two coating thicknesses (10 μm and 50 μm), solidification cracking occurred in most of the Ni–P-coated Cu–steel welds. For the Ni–P-coated welds, solidification cracking was significantly aggravated when the coating thickness increased from 10 μm to 50 μm. In contrast, for the pure Ni-coated Cu–steel welds, solidification cracking was effectively suppressed for both 10 μm and 50 μm coatings. That is, regardless of the beam-scanning pattern applied, pure Ni-coated Cu–steel welds consistently produced crack-free welds, demonstrating superior resistance to solidification cracking.

The weld mushy-zone range at local microregions at the solidification cracking site was evaluated using Thermo-Calc calculations. In the Ni–P 10 μm coated Cu–steel welds, the concentration of P in the remaining liquid phase increased to approximately 8.8 mass%, and the calculated mushy-zone width reached a maximum of 869 K, indicating an extremely wide solidification temperature range. In contrast, the pure Ni 50 μm coated Cu–steel welds exhibited a very low P concentration of approximately 0.1 mass% in the remaining liquid at the final stage of solidification completion. The resulting solidification temperature range was relatively narrow, within approximately 200–400 K, indicating substantially reduced susceptibility to solidification cracking. Accordingly, cracking was not observed in the microstructure, and the corresponding shear-tensile tests exhibited the highest load-bearing capacity and displacement among all evaluated conditions.

These findings indicate that localized enrichment of P substantially widens the solidification temperature range, thereby increasing the susceptibility to solidification cracking. Conversely, the suppression of P segregation narrows the mushy-zone range and mitigates solidification cracking. Therefore, non-addition of P in the coating layer is strongly recommended for ensuring the structural integrity of Cu–steel dissimilar welds.

Based on the findings of this study, the 50 μm pure Ni coating is identified as the most suitable coating condition for cylindrical lithium-ion battery busbar laser welding. This coating not only suppresses solidification cracking and enhances joint strength (from 154.8 kgf for Ni–P 50 μm to 218.5 kgf for pure Ni 50 μm under the Spiral beam pattern), but also improves laser-beam absorption by reducing the surface reflectivity of Cu. Accordingly, under the laser-welding parameters used in this study, a Ni coating thickness of approximately 50 μm can be considered the recommended minimum for ensuring crack resistance and high load-bearing capacity, while thinner Ni layers provide only partial improvement and require careful optimization of welding conditions.

## Figures and Tables

**Table 1:** Chemical composition of materials used (mass%).

Materials	Cu	Fe	C	Si	Mn	P	S	Pb	Sn	Ni
Copper (C1100P)	Bal.	0.0043	-	-	-	-	-	0.0005	0.0010	0.0008
Mild steel (SS275)	-	Bal.	0.0020	0.0010	0.0990	0.0109	0.0038	-	-	-

**Table 2:** Plating methods for Cu surface and their key parameters.

Plating Method	Bath Composition (g/L)	pH	Temperature (°C)	Current Density (A/dm^2^)	Deposition Rate (µm/hr)
Electroless Plating (Ni–P coating)	NiSO_4_: 24.5–25.5	4.8–5.3	85–95	-	10–15
Electroplating (pure Ni coating)	Nickel sulfamate: 360–400	3.5–4.5	45–55	3–8	30–35
	Boric acid: 30–40				

**Table 3:** Specific conditions for single-mode fiber laser welding.

Laser Source	Laser Power (W)	Defocus Distance (mm)	Beam Diameter at Focal Point (μm)	Scan Speed (mm/s)	Heat Input (J/mm)	Beam Pattern	Wobble Frequency (Hz)	Wobble Amplitude (mm)	Pitch (mm)
Single mode Fiber laser (Wave -length: 1068 nm)	2000	0	38	1100	1.82	Linear	-	-	-
						Spiral	-	-	-
						Wobble + Spiral	4000	0.1	0.28

**Table 4:** Standard deviation and 95% confidence intervals of shear-tensile strength under different coating thicknesses and laser beam-scanning patterns.

Coating Condition of Cu	Laser Beam Pattern	Mean ± Standard Deviation (kgf)	95% Confidence Intervals (kgf)
Ni–P 10 µm	Linear	128 ± 4.67	123.7–132.3
	Spiral	193 ± 5.07	188.3–197.7
	Spiral + Wobble	218 ± 7.21	211.3–224.7
Ni–P 50 µm	Linear	77 ± 6.99	70.5–83.5
	Spiral	154 ± 6.27	148.2–159.8
	Spiral + Wobble	95 ± 4.95	90.4–99.6
Ni 10 µm	Linear	103 ± 9.18	94.5–111.5
	Spiral	197 ± 12.0	185.9–208.1
	Spiral + Wobble	188 ± 16.2	173.0–203.0
Ni 50 µm	Linear	99 ± 5.93	93.5–104.5
	Spiral	219 ± 3.70	215.6–222.4
	Spiral + Wobble	190 ± 15.9	175.3–204.7

**Table 5:** Representative chemical compositions extracted from local points within the fusion zone of Ni–P- and Ni-coated Cu–steel welds (input parameters for the Thermo-Calc mushy-zone calculation).

10 μm Ni–P Coated Cu–Steel Welds (Linear Pattern)		50 μm Ni–P Coated Cu–Steel Welds (Linear Pattern)		10 μm Ni Coated Cu–Steel Welds (Spiral Pattern)		50 μm Ni Coated Cu–Steel Welds (Spiral Pattern)	
Cu	69.9661	Cu	55.7528	Cu	43.0418	Cu	29.4866
Fe	28.0908	Fe	35.7565	Fe	51.9342	Fe	60.0236
C	0.5037	C	0.5203	C	0.4060	C	0.3435
Mn	0.1077	Mn	0.0132	Mn	0.1596	Mn	0.1264
Si	0.0000	Si	0.0115	Si	0.0048	Si	0.0000
Ni	1.1010	Ni	7.3400	Ni	4.3716	Ni	10.0149
P	0.2307	P	0.6057	P	0.0820	P	0.0051

**Table 6:** Input parameters used for the Thermo-Calc mushy-zone calculations.

Calculation Method	Parameters	Values
Scheil with back diffusion in the primary phase	Scanning speed (mm/s)	1100
	Cooling rate (K/s)	10,000
	Secondary dendrite arm spacing (m)	0.000001
	Solidification complete ratio (%)	99

## Data Availability

The original contributions presented in this study are included in the article. Further inquiries can be directed to the corresponding author.
